# Barriers and facilitators to exercise engagement during lung cancer treatment: a scoping review

**DOI:** 10.1007/s10552-026-02177-6

**Published:** 2026-05-26

**Authors:** Matthew Beggs, Joanne Reid, Gerard G Hanna, Gillian Prue

**Affiliations:** 1 School of Nursing and Midwifery, Queens University Belfast, Belfast, Northern Ireland; 2 Trinity St. James’s Cancer Institute, Trinity College Dublin, Dublin, Ireland; 3 St. Luke’s Radiation Oncology Network, St. Luke’s Hospital Rathgar, Dublin, Ireland

**Keywords:** Scoping review, Cancer therapy, Lung cancer, Fitness, Well-being, Oncology

## Abstract

**Purpose:**

Evidence suggests that exercising through lung cancer (LC) treatment may improve outcomes by modulating the tumor microenvironment and reducing hypoxia. However, recruitment and adherence challenges persist, limiting research progress and real-world implementation. Therefore, this scoping review aimed to explore the barriers and facilitators that influence exercise participation among individuals with LC during cancer treatment.

**Methods:**

This ScR followed the JBI ScR methodology and the PRISMA-ScR checklist, using the participants–concept–context framework to define inclusion and exclusion criteria. The data were analyzed via qualitative content analysis with the themes presented in a narrative summary supported by tables and figures.

**Results:**

The initial search yielded *n* = 24,242 results, of which twenty-five studies (*n* = 25) were included for data extraction. The analysis identified 31 barriers and 42 facilitators grouped into five themes: physiological status; intrapersonal factors; program design and delivery; practical, logistical, and interpersonal factors; and professional/clinical considerations. Fatigue was the most frequently cited barrier, followed by intense periods of other treatment side effects experienced throughout treatment cycles. Conversely, personalized exercise programs and peer support were the most cited facilitators. Only two studies reported fatigue and muscle soreness as adverse events.

**Conclusion:**

People with LC face many barriers that make exercising during treatment challenging. However, this review presents a list of exercise facilitators that researchers and service providers could adopt to inform the development of patient-centered exercise programs that encourage engagement, potentially leading to improved cancer treatment outcomes.

**Supplementary Information:**

The online version contains supplementary material available at 10.1007/s10552-026-02177-6.

## Introduction

Since MacVicar and Winningham’s landmark study on exercise during chemotherapy [1], exercise oncology research has expanded substantially to encompass multiple cancer types, including breast, lung, colorectal, and prostate cancer [2–6]. This growing evidence base has informed international exercise guidelines for cancer survivors [3, 6], with a recent systematic review conducted by the American Society of Clinical Oncology (ASCO) [4] concluding that healthcare providers should recommend exercise during curative cancer treatment.

Lung cancer (LC) remains the leading cause of cancer-related mortality [7, 8] and is responsible for approximately 18.7% of all global cancer deaths [9]. Furthermore, five-year survival rates remain low, averaging 10–20%, depending on the country [8–10]. Although evidence on the impact of exercise on cancer treatment outcomes is limited [3, 4], preclinical and mechanistic studies suggest that exercising during treatment may improve outcomes for LC survivors through several mechanistic pathways [11–16]. These include modulation of the tumor microenvironment to reduce inflammation, increased infiltration of natural killer cells, and improved tumor oxygenation. For example, preclinical studies have demonstrated that exercise may increase tumor sensitivity to radiotherapy (RT) by reducing tumor hypoxia (TH) [13–15]. This is relevant to LC survivors, as TH is estimated to occur in 50–80% of non-small cell lung cancers (NSCLCs) [14], which can increase resistance to RT in up to three-thirds of tumors [16]. However, the clinical significance of these findings remains debated [17–22], as systematic reviews cite methodological heterogeneity and inconsistent results, questioning the robustness of evidence [20]. Furthermore, the limited availability of clinical studies means that preclinical findings have not been translated into real-world practice, leaving this area largely hypothetical [18, 21].

Despite this debate, exercise has been associated with improving the physical and psychological resilience of LC survivors, providing benefits such as improved fitness and quality of life (QoL), which may contribute to improving treatment outcomes [17, 18]. Therefore, considering its potential, there is a clear need for rigorous, comparative trials to study the potential therapeutic efficacy of exercise for the treatment of LC [18–23]. However, LC research consistently highlights recruitment and adherence challenges due to physical and psychological barriers such as symptom burden, smoking-related stigma, and negative attitudes toward exercise [18, 22]. This is concerning, as progress in LC exercise research will remain limited until these barriers are better understood and addressed. Therefore, mapping the barriers and facilitators influencing exercise engagement among people with LC during treatment is essential. Such insights could guide the development of patient-centered exercise programs that may encourage LC survivors to engage with exercise research or services during cancer treatment [18, 21].

## Materials and methods

### Review question and objectives

*Aim*: To explore the barriers and facilitators that influence exercise participation among individuals with LC during cancer treatment.

*Objectives*:

1. To explore the barriers and facilitators influencing exercise engagement among individuals living with LCs during treatment, as perceived by patients, family members, caregivers, and healthcare professionals (HCPs).
2. To identify any reported safety concerns or adverse events associated with exercise interventions during LC treatment.
3. To map the identified barriers and facilitators in a coherent and accessible format to inform future research and intervention design.

### Review design

A scoping review (ScR) was conducted adhering to the Joanne Briggs Institute (JBI) methodological guidance for conducting scoping reviews [24]. The JBI is an international collaboration of scientific experts committed to improving research methodology and has developed the most recent ScR guidelines to support the rigorous conduct of ScR’s, thereby improving transparency and trustworthiness [24, 25]. In addition, the Preferred Reporting Items for Systematic Reviews extension for Scoping Reviews (PRISMA-ScR) checklist was followed to ensure the rigor and transparency [25, 26] provided in Online Resource 1.

### Search strategy

An initial search strategy was developed in consultation with a subject librarian to search three databases: EBSCO Host, PubMed, and OVID. A preliminary search identified key terms and informed the strategy based on three concepts: lung cancer, exercise, and barriers/facilitators. Boolean operators (‘OR’ within concepts and ‘AND’ between concepts) were applied. Additional sources were sought by screening the reference lists of the included articles and gray literature. The full search strategy is provided in online resource 2. The search was executed on 16th September 2025.

### Eligibility criteria

In accordance with JBI’s methodology, the Participants, Concept, Context framework was used to predefine the inclusion and exclusion criteria [25].

#### Population

- Adults (aged 18 and over) are diagnosed with LC
- All types, grades, and stages of LC were considered.
- Informal carers, family members, and friends of individuals diagnosed with LC
- HCPs, exercise professionals, researchers, and other personnel involved in facilitating exercise research or services for people with LC

#### Concept

The principal concept is the identification of barriers and facilitators to exercise among people with LC. Within this ScR, barriers are defined as factors that prevent, limit, or obstruct exercise engagement, whereas facilitators are defined as factors that enable or encourage engagement [27].

#### Context

This ScR focuses on studies exploring exercise during LC treatment. For clarity, cancer treatment refers to modalities such as radiotherapy (RT) and systemic anticancer therapies (SACTs) [28]. Studies involving exercise before and after surgery were excluded as they are considered prehabilitative or rehabilitative exercise. Exercise refers to voluntary, physical activity above resting levels, with defined intensity, duration, and frequency [29]. Exercise as standalone programs or as part of multimodal interventions was included, provided that exercise-related data could be extracted. Qualitative studies without exercise were included if their objectives aligned with this ScR.

Studies were not limited by country, date, or design. Systematic and other literature reviews were also considered for inclusion. Studies were excluded if they:

- Involved noncancer populations (e.g., mixed participant groups).
- Focused on nonexercise interventions, such as specialist breathing exercises or meditation programs.
- Were research protocols that had not published results.
- Conference abstracts or citations without full-text access.
- Not written in English as the primary language.

### Study selection

**Fig. 1:**
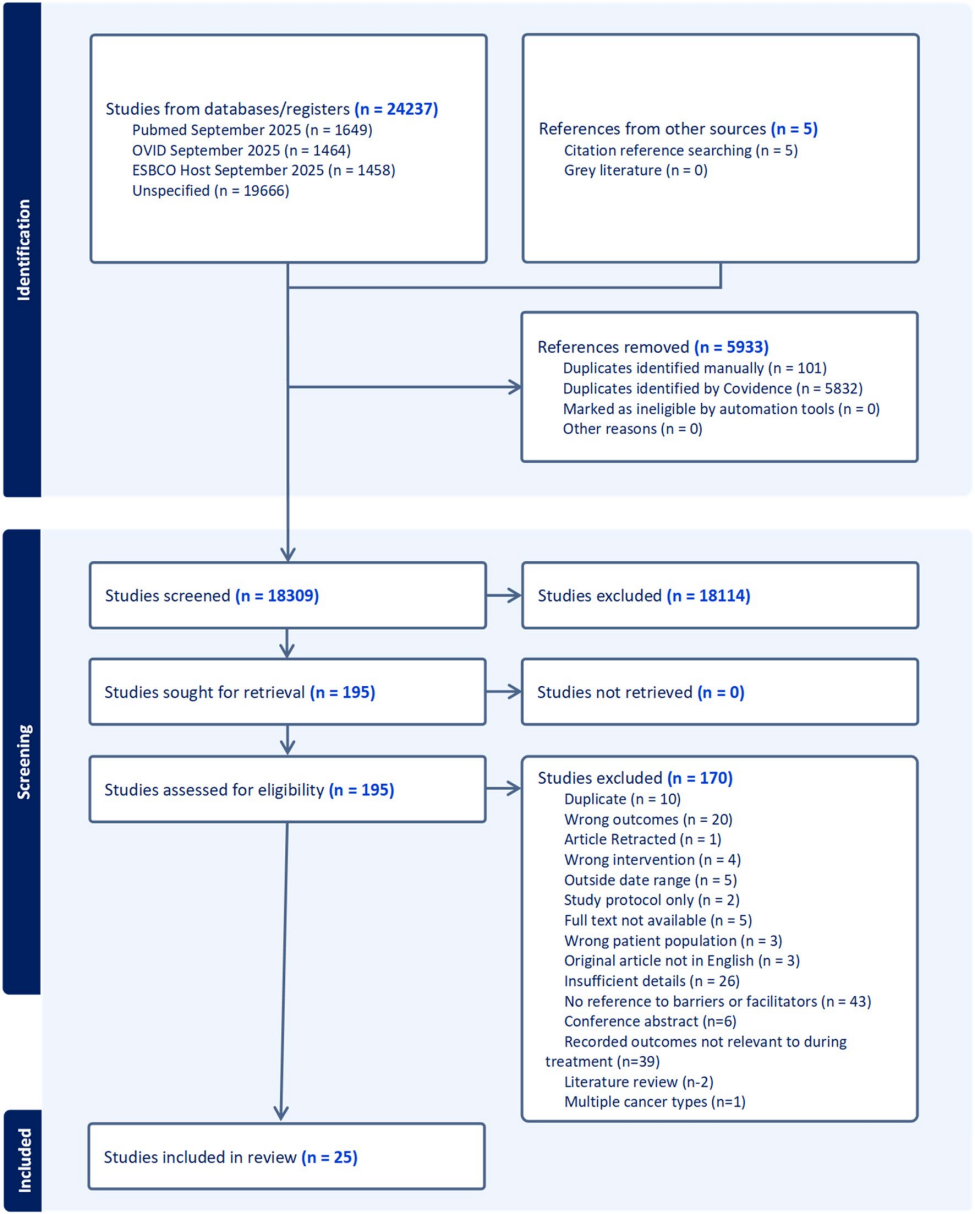
Prisma diagram

Citations were imported into Covidence for title/abstract screening by MB, followed by full-text review. Two secondary reviewers (GP and JR) independently screened 5% of the articles each (10% total), aiming for ≥ 90% agreement. Discrepancies were resolved through discussion. The search results are summarized in a PRISMA flow diagram (Fig. 1).

### Data extraction

MB extracted data via a table adapted from JBI and PRISMA-ScR templates [24, 26] to align with this ScR’s objectives, with input from two independent researchers (GP and JR). Disagreements were resolved through discussion. The extracted variables included author, year, country, design, sample characteristics, intervention type and setting, adverse events, and barriers/facilitators, as illustrated in Table 1. The data were managed in Microsoft Excel. The data extraction sheet (Table 1) is available in online resource 3.

**Table 1:** Details of the included studies (*n* = 25)

Author, year	Country	Research design	Participants	Treatment	Exercise program details			Adverse events	Codes	
					Type	Setting	Description		Barriers	Facilitators
Wang et al*. (*2025)	Canada	Prospective, single-arm, feasibility study	*n* = 26 NSCLC & SCLC	Targeted therapy, Chemotherapy, Immunotherapy, * Or combination	Combination of aerobic and resistance exercises	Home-based	Virtual, supervised, group exercise: Twice per week for 12 weeks duration (3 months) Each session included: 10-min warm-up + 30-min exercise circuit (2–3 sets) + 5–10-min cool down Content: Functional movements targeting major upper and lower body muscle groups including combination of aerobic (e.g., marching on the spot) and resistance exercises using bands Progression: Intensity guided by Borg RPE scale, starting at 10–12/20 and progressing to 12–14/20 over 12 weeks Individualization: Exercises adapted to participants’ functional levels (e.g., seated, supported standing, or unsupported standing) Based on best practices for older adults’ functional mobility Participants remained visible on camera for real-time feedback and correction	No major adverse events related to the intervention were recorded. Minor adverse events such as fatigue and muscle soreness were tracked by the instructor as part of attendance and adherence tracking, however without a more systematic approach, this may have led to underreporting, limiting understanding of the study’s safety profile	1. Treatment Scheduling 2. Travel/distance 3. Cancer Treatment 4. Fatigue 5. Feeling unwell 6. Unsupervised exercise	1. Virtual exercise 2. Supervised exercise 3. Group exercise 4. Accountability measures
Quadflieg et al. (2025)	Belgium	Longitudinal observational Study	*n* = 63 NSCLC	Chemotherapy, Radiotherapy, Surgery	No exercise intervention—Observational study, monitoring the impact of cancer treatment on PA	n/a	n/a	n/a	1. Cancer treatment 2. Comorbidities	1. Personalized programs
Lee et al.* (*2025)	Korea	Randomized control trial	*n* = 65 Stage II NSCLC: 57 SCLC:8	Radiotherapy	Combinations of aerobic exercise, resistance training alongside breathing exercises, coughing techniques, and chest wall stretching	Hospital based	Supervised: Throughout radiotherapy (26—40 days) 1 h session, 2—3x/week	None reported	None reported	1. Treatment/appointment alignment
Presley et al. (2024)	USA	Single-arm feasibility study	*n* = 11 Unresectable stage III/IV NSCLC or SCLC	Chemotherapy	Combination of aerobic exercise, resistance training, full body stretches, and relaxation	Combination of home and hospital based	All sessions Supervised In person (Once per week) - 10 min warm-up - Core and resistance training (bands) - Full body stretches Virtual (× 2 sessions per week): Individualized program with same equipment Duration 3 months (12 weeks)	None reported	1. Use of Technology 2. Travel/distance 3. Lack of time 4. Treatment scheduling 5. Fatigue 6. Cancer treatment 7. Advanced disease 8. Life stressors	1. Good baseline functional scores 2. Supervised Exercise 3. Treatment/appointment alignment 4. Virtual exercise
Kiss et al. (2024)	Australia	Qualitative study	*n* = 18 NSCLC: 14 SCLC:4	Chemotherapy	No exercise program—qualitative interviews only	n/a	n/a	n/a	1. Cancer treatment 2. Lack of information/education 3. Nausea and vomiting 4. Fatigue 5. Esophagitis 6. Lack of resources	1. Experienced positive benefits 2. Adaptable programs 3. HCP/Professional support 4. Group exercise 5. Peer support 6. Professional advocacy 7. Personalized programs 8. Trust between professionals 9. Accountability measures 10. Clear, practical, and affirming educational material
Hou et al. (2024)	China	Cross-sectional study	*n* = 208 NSCLC: 175 SCLC: 33	Chemotherapy	No exercise program – surveys and questionnaires only	n/a	n/a	n/a	1. Nausea & Vomiting 2. Lack of appetite 3. Fatigue 4. Comorbidity 5. Pain/discomfort 6. Lack of variety 7. Fixed intensity	1. Personalized programs 2. Light Intensity 3. Adaptable program
Bowman et al. (2024)	Australia	Qualitative study	*n* = 15 LC nonspecific	Chemotherapy, Radiotherapy, Surgery, Immunotherapy, Targeted therapy (EGFR Inhibitor), * Or combination	No exercise program—qualitative interviews only	n/a	n/a	n/a	1. Cancer treatment 2. Medications	1. Personalized programs 2. Peer support 3. HCP/Professional support 4. Exercise monitoring and progressing
Bloch et al. (2024)	Denmark	A Secondary analysis of a randomized controlled trial	*n* = 228 Advanced NSCLC: 192 SCLC: 36	Chemotherapy, radiotherapy	Combination of high-intensity resistance training and aerobic exercise	Hospital based	Group, Supervised, in-person exercise, twice/week for 12 weeks	No serious adverse events associated with the intervention were reported	1. Anxiety/depression 2. Low baseline functional scores	1. Good baseline functional scores, 2. Home-based/community programs 3. Remote exercise supervision 4. Flexible scheduling
Voorn et al. (2023)	Netherlands	Case study	*n* = 1 Advanced NSCLC	Chemotherapy, radiotherapy	Combination of Aerobic, resistance, and breathing exercises	Home-based	Total duration: 32 weeks	Not specified	1. Fatigue 2. Feeling unwell 3. Shortness of breath	1. Perceived benefits 2. Maintenance of ADL’s 3. Peer support 4. Supervision 5. HCP support 6. Adjustable Program 7. Treatment specific programs 8. Personalized programs 9. Emotional support
Voorn et al*.* (2022)	Netherlands	Feasibility, proof-of-concept study	*n* = 6 Advanced NSCLC	Chemotherapy	Combination of Aerobic, resistance, and breathing exercises	Home-based	Unsupervised with × 1 supervised session every 2 weeks (1 of 10) Participants performed: - Aerobic exercise 5 times/week for 30 min (functional exercises e.g., walking, cycling, stair climbing) - Resistance exercises × 3/week - Breathing exercises twice daily Total duration: 13—18 weeks	There were no dropouts or adverse events because of the rehabilitation program	1. Fatigue 2. Anxiety/depression 3. Pain 4. Shortness of breath 5. Treatment Scheduling 6. Unsupervised exercise	1. Adaptable programs 2. Supervision 3. Peer support 4. HCP/Professional Support 5. Home-based exercise 6. Personalized programs 7. Multimodal programs 8. Light Intensity
Swan et al. (2021)	UK	Qualitative Interviews	*n* = 9 Lung cancer: 3 Mesothelioma:10 Informal carers/family members: 9 HCP: 9	Chemotherapy, Immunotherapy	No exercise program—qualitative interviews only	n/a	n/a	n/a	1. Lack of information/education 2. Lack of referable services 3. Fear 4. Shock of diagnosis 5. Cancer Treatment 6. Infection risk 7. Comorbidities 8. Lack of referral pathways 9. Lack of resources 10. High clinical workload 11. Anxiety/depression	1. Clear, practical and affirming educational material 2. Address safety concerns 3. Peer support 4. Group exercise 5. Treatment specific programs 6. Personalized programs 7. Adaptable programs 8. Multimodal programs 9. Carer Support
Ester et al. (2021)	Canada	Feasibility study: single arm	*n* = 10 Advanced lung cancer	Chemotherapy, Targeted therapies (Tyrosine Kinase Inhibitor & Immune Checkpoint Inhibitors)	Combination of light aerobic walking with resistance training	Home-based	Combination of group and individual exercise sessions. Also included both supervised and unsupervised. Duration for 12—14 weeks In-person: twice per week Hom based: 2—4 days of light aerobic (walking) exercise and 1—2 days of resistance training	No adverse events related to the intervention were reported	1. Lack of peer support 2. lack of motivation	1. Personalized exercise programs 2. Optimized duration 3. Enjoyment/fun 4. HCP/Professional support 5. Peer support 6. Home-based/community programs 7. Flexible scheduling 8. Supervised 9. Group-based
Edbrooke et al. (2020)	Australia	(Mixed method) An experimental study with qualitative interviews	*n* = 92 Inoperable NSCLC	Chemotherapy, Radiotherapy	Combination of moderate intensity aerobic exercise with resistance training	Home-based	Individual, unsupervised, 2—3 times per week for 8 weeks	No serious adverse events related to the program occurred	1. Hospital-based exercise 2. Fatigue, Dyspnea, 3. Dizziness, 4. Diarrhea, 5. Pain 6. Lack of appetite, 7. Coughing 8. Pain, 9. Infection	1. Flexible scheduling 2. Peer support 3. HCP/Professional support 4. Personalized exercise programs 5. Home-based/community programs 6. Requires no specialist equipment 7. Exercise monitoring & progression 8. Motivation 9. Accountability measure
Granger et al. (2019)	Australia	Qualitative study	*n* = 7 Lung cancer	All treatments	No exercise program—qualitative interviews only	n/a	n/a	n/a	1. Limited exercise history/Sedentary lifestyle 2. Comorbidities 3. Adverse weather conditions 4. Fear 5. Peer discouragement 6. Employment 7. Travel/location 8. Financial cost 9. lack of information. education & support 10. Boring/unenjoyable 11. fatigue 12. Shortness of breath 13. Pain 14. Depression/Anxiety 15. lack of time 16. Use of technology only	1. Previously experienced positive benefits 2. Maintenance of ADL’s 3. Flexible scheduling 4. Exercise monitoring & progression 5. HCP/Professional support 6. Group-based 7. Exercise History 8. Enjoyment/fun 9. Accountability 10. Personalized exercise programs 11. Physical Activity counseling/Behavior change support 12. Hospital-based exercise
Payne et al. (2018)	UK	Qualitative exploration study	*n* = 14 Advanced NSCLC: 8 HCP:6	Chemotherapy	No exercise program—qualitative interviews only	n/a	n/a	n/a	1. Disbelief in benefits	1. Perceived unharmful 2. Treatment/appointment alignment 3. Short Duration 4. Clear and practical educational material 5. Exercise monitoring and progressing 6. Personalized programs 7. HCP/Professional support 8. Achievable 9. Motivation 10. Experienced positive benefits 11. Perceived Benefits 12. Peer support 13. Flexible Scheduling
Oliver et al. (2018)	France	Prospective uncontrolled observational pilot study	*n* = 71 Unresectable LC or Malignant pulmonary mesothelioma	Chemotherapy	Combination of endurance training (cycle erg), muscle strengthening exercises (resistance bands/weights), and activities of daily living (stair climbing/walking)	Home-based	Unsupervised 30—45 min of exercise at least 5 days/week	No potential adverse events related to PR activities were reported	1. Lack of motivation 2. Shock of diagnosis 3. Fatigue 4. Treatment scheduling 5. Advanced Disease	1. Physical Activity counseling/Behavior change support 2. Home-based exercise
Ni et al. (2018)	China and Australia	Observational Cohort Study	*n* = 161 Newly Diagnosed LC	All treatments	No exercise program—surveys and questionnaires only	n/a	n/a	n/a	1. Shock of diagnosis 2. Treatment scheduling	1. Cultural habits for transportation 2. Treatment specific programs
Karvinen et al. (2016)	USA	Qualitative Survey	*n* = *43* Lung cancer	Chemotherapy	No exercise program—qualitative interviews only	n/a	n/a	n/a	1. Fatigue 2. Pain 3. Feeling unwell 4. Lack/inconvenience of facilities	1. Convenient locations 2. Peer support 3. Feeling well/energy 4. Light intensity 5. Physical activity counseling/Behavior change support 6. Shock of diagnosis “teachable moment” 7. Perceive benefits 8. Home-based/community programs
Kartolo et al. (2016)	Canada	Cross-sectional study – survey	*n* = 60 Inoperable metastatic lung cancer	All treatments excluding surgery	No exercise program—surveys and questionnaires only	n/a	n/a	n/a	1. Comorbidities 2. Travel/distance 3. Feeling unwell 4. Adverse weather conditions	1. Perceive benefits 2. Achievable 3. Peer support 4. HCP/Professional support 5. Exercise history 6. Convenient locations 7. Enjoyment/fun 8. Flexible scheduling
Mas et al. (2015)	France	Exploratory study	*n* = 5 Advanced NSCLC	Chemotherapy	No exercise program—qualitative interviews only	n/a	n/a	n/a	1. Fatigue 2. Pain 3. Nausea & vomiting 4. Infection 5. Constipation 6. Neuropathy 7. Anxiety/depression 8. Shock of diagnosis 9. Comorbidities 10. Fear 11. Unemployment 12. Peer discouragement 13. Adverse weather conditions 14. Lack of information/education	1. Peer support 2. HCP/Professional support 3. Exercise History 4. Activities which involve focus strategy/technique 5. Perception and experience of deterioration 6. Exercise Variety
Leach et al. (2015)	Canada	Cross-sectional study: retrospective survey design	*n* = 66 Lung cancer	Chemotherapy, Radiotherapy	No exercise program—surveys and questionnaires only	n/a	n/a	n/a	1. Cancer Treatment	1. Personalized exercise programs
Kuehr et al. (2014)	Germany	Feasibility study	*n* = 40 Advanced NSCLC	Chemotherapy with radiotherapy, Chemotherapy only	Combinations of endurance and resistance training	Combination of home and hospital based; Combination of inpatient and outpatient treatment plans	Inpatient group Individual, supervised and unsupervised exercise: Supervised – × 3 session per week face to face in hospital Unsupervised – × 2 sessions per week at home Outpatient group X3 unsupervised exercise sessions with once weekly telephone support	No adverse event relating to the exercise intervention program occurred	None documented	1. Personalized exercise programs 2. Home-based/community programs 3. HCP/Professional support
Henke et al*.* (2014)	Germany	Randomized controlled trial	*n* = 46 Advanced NSCLC	Chemotherapy	Combination of endurance training, strength training, breathing techniques, and conventional physiotherapy	Combination of home and hospital based	Endurance: 6-min hall walking + 2-min stair walking 5 days/week Strength training unspecified	None reported	1. Cancer treatment 2. Advanced disease 3. Travel/distance 4. Low baseline functional scores	1. Exercise monitoring and progressing 2. Experienced positive benefits 3. Exercise history 4. Good baseline functional scores
Adamsen et al. (2012)	Denmark	Qualitative study	*n* = 15 NSCLC: 13 SCLC: 2	Chemotherapy, Radiotherapy	Combination of resistance and aerobic exercises with relaxation	Combination of home and hospital based	Combination of group and individual exercise sessions. Also included both supervised and unsupervised. Duration for 6 weeks	None reported	1. Lack of self-discipline 2. Limited exercise history 3. Shock of diagnosis 4. Pain/discomfort 5. Home-based exercise 6. Disbelief in benefits 7. Fatigue 8. Shortness of Breath 9. Exercising alone 10. Treatment scheduling 11. Infection	1. Shock of diagnosis “teachable moment” 2. Group exercise 3. Supervised exercise 4. Trust between professionals 5. Personalized programs
Andersen et al. (2011)	Denmark	Feasibility study	*n* = 24 NSCLC: 19 SCLC (+ Mixed): 5	Chemotherapy, Radiotherapy, Targeted therapies (Tyrosine Kinase Inhibitor)	Combination of walking and high-intensity interval training	Combination of home and hospital based	Combination of group and individual exercise sessions. Also included both supervised and unsupervised. Duration for 7 weeks	low (undefined)	1. Fixed exercise programs 2. Anxiety/depression 3. Fatigue 4. Pain/discomfort 5. Comorbidities 6. Resuming employment 7. Fear	1. Group-based exercise 2. Supervised exercise (in-person or remote) 3. Peer support

^*^ Or combination – refers to some participants may have received combinations of the listed treatments document within the study

*LC* lung cancer, *SCLC* Small-cell lung cancer, *NSCLC* Non-small cell lung cancer, *HCP* Healthcare professional, *ADL’s* Activities of daily living, *RPE* Rate of perceived exertion, *PR* Pulmonary rehabilitation

### Data analysis and presentation

Data analysis was conducted via qualitative content analysis in three distinct phases: [30]

1. Preparation phase

The unit of analysis comprised individual words or complete sentences from the included articles. Both manifest and latent content were considered, as barriers and facilitators are not always explicit, particularly in quantitative studies that use terms such as “enable” or “limitation”. Source characteristics, the frequency of barriers and facilitators, and adverse events were summarized via descriptive statistics. An inductive approach guided the analysis.

(2) Organization phase

Following data familiarization, MB performed line-by-line open coding, condensing sentences into codes grouped under barriers, facilitators, and adverse events. Codes were iteratively reviewed, merged, and organized into subcategories on the basis of emerging themes. The coding framework was refined during extraction and finalized through consensus among reviewers (MB, GP, JR).

(3) Reporting

A narrative summary describes the themes within each category (barriers, facilitators, adverse events) and is complemented by visual representations, such as figures and tables, to convey findings.

## Results

### Search

The initial search identified 24,242 studies. After *n* = 5,933 duplicates were removed, *n* = 18,309 were subjected to title and abstract screening. A total of 195 studies, of which n = 25 were selected for data extraction, were subjected to full-text screening. Figure 1 displays the PRISMA flow chart, illustrating the selection process and reasons for exclusion.

### Details of the study characteristics

All the study characteristics are presented in Table 1; however, the following provides the context of the included studies.

#### Place of publication

Within this ScR, five studies were conducted in Australia (*n* = 5), four in Canada (*n* = 4), and 3 in Denmark (*n* = 3). Two studies were conducted in the United States of America (USA), Germany, France, the United Kingdom (UK), the Netherlands, and China (*n* = 2). Finally, one study was conducted in Korea (*n* = 1) or Belgium (*n* = 1).

#### Research methodology

The included studies employed diverse methodologies (Table 2). Thirteen of the 25 studies incorporated an exercise intervention [31–43], whereas 12 used qualitative approaches such as surveys and interviews [44–55].

**Table 2:**
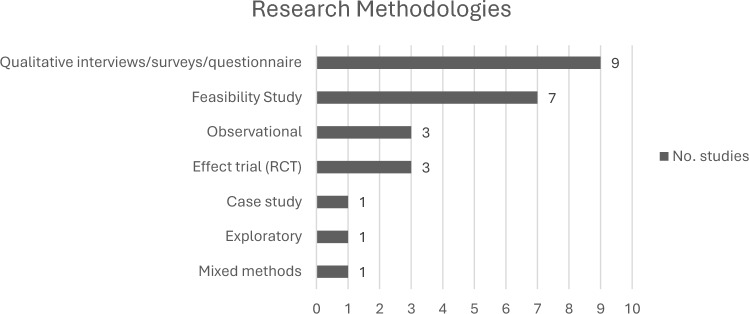
Methodology of included studies

#### Intervention characteristics

Among the 13 studies that included an exercise program, six combined supervised and unsupervised sessions [31–33, 36–38], six provided supervised exercise only [34, 39–43], and one offered unsupervised exercise only [35]. Mos^36^t programs are individual-based [33–38, 40–42], whereas five incorporate group sessions [31, 32, , 44]. Eleven studies delivered home-based programs, six exclusively [33–38, 43] and five combining home and hospital settings [31–33, 40, 41]; only two were hospital-based [39, 43]. Nearly all programs (24 out of 25) included both aerobic and resistance exercises, with one study offering aerobic exercise alone [31]. A summary is provided in Table 3.

**Table 3:** Summary of exercise program characteristics included in this review

	Level of supervision		Methods of exercise		Place of exercise		Types of exercise	
Study	Unsupervised	Supervised	Individual	Group	Home-based	Hospital-based	Resistance	Aerobic
Andersen et al*.,* 2011 [31]	**x**	**x**	**x**	**x**	**x**	**X**		**x**
Adamsen et al., 2012 [32]	**x**	**x**	**x**	**x**	**x**	**X**	**x**	**x**
Bloch et al*.,* 2024 [39]		**x**		**x**		**X**	**x**	**x**
Edbrooke et al*.,* 2020[35]	**x**		**x**		**x**		**x**	**x**
Ester et al*.,* 2021[36]	**x**	**x**	**x**	**x**	**x**		**x**	**x**
Kuehr et al*.,* 2014 [33]	**x**	**x**	**x**		**x**	**X**	**x**	**x**
Oliver et al*.,* 2018		**x**	**x**		**x**		**x**	**x**
Voorn et al*.,* 2022	**x**	**x**	**x**		**x**		**x**	**x**
Voorn et al*.,* 2023 [38]	**x**	**x**	**x**		**x**		**x**	**x**
Henke et al., 2014[40]		**x**	**x**		**x**	**X**	**x**	**x**
Lee et al*.,* 2025 [42]		**x**	**x**			**X**	**x**	**x**
Presley et al*.,* 2024 [41]		**x**	**x**		**x**	**X**	**x**	**x**
Wang et al*.,* 2025 [43]		**x**		**x**	**x**		**x**	**x**

### Barriers and facilitators

The analysis followed line-by-line coding. The codes were initially categorized as either barriers or facilitators. In total, n = 31 barriers and n = 42 facilitators were identified. Following the merging of the initial coding, five themes emerged to group the coded barriers and facilitators. These include:

1. Physiological status
2. Intrapersonal factors
3. Program design and delivery determinants of engagement
4. Practical, logistical and interpersonal factors
5. Professional and clinical considerations

### Theme 1: Physiological status

This theme reflects physical symptoms and characteristics that limit exercise participation during LC treatment.

### Barrier

Fifteen physical symptoms were identified as barriers to exercise. Fatigue was the most frequently reported form of fatigue (*n* = 11) [32, 34, 35, 37, 41, 43, 45, 47, 50, 53, 54] [27, 29, 30, 32, 36, 38, 40, 42, 45, 48, 49], followed by pain or discomfort (n = 7) [27, 30, 32, 40, 42, 45, 48] and shortness of breath (n = 4) [27, 30, 32, 45]. Four studies [32, 35, 45, 51] reported that fear of infection led to exercise-avoidant behaviors, such as avoiding public exercise facilities, whereas actual infections prevented participation. Other issues included nausea and vomiting (n = 3) [45, 53, 54], lack of appetite (n = 2), esophagitis (n = 1) [54], neuropathy (n = 1) [45], constipation (n = 1) [45], coughing (n = 1) [35], and generally feeling unwell (n = 3) [43, 46, 47]. Three (n = 3) studies cited advanced disease as a physical condition that limits exercise engagement [34, 40, 41], which is consistent with two studies (n = 2) reporting low baseline functional scores [39, 40] as barriers.

### Facilitators

Two studies cited physical conditions that facilitated exercise engagement, including good baseline functional scores (n = 3) [39–41] and feeling well or having energy (n = 1) [47], referring to the absence of symptoms and side effects.

### Theme 2: Interpersonal factors

This theme reflects individual-level barriers and facilitators, including emotional responses (e.g., fear, anxiety) and how personal perceptions, attitudes, and prior exercise experiences shape engagement [56].

### Barriers

Ten barriers were identified, the most prominent being anxiety or depression (n = 5) [37, 39, 45, 50, 51] and the shock of diagnosis (n = 5) [32, 34, 45, 48, 51]. These factors have been reported to cause psychological distress, causing participants to deprioritize exercise. Four studies cited a lack of information or education [45, 50, 51, 54], often due to insufficient knowledge or poor communication from HCPs. However, disbelief in exercise benefits (n = 2) [32, 49] suggests that education alone may not improve engagement, as some participants remained unconvinced about the benefits of exercise, despite being informed by the researchers. Other barriers included fear of exercise causing harm (n = 3) [45, 50, 51] and low internal drive, such as lack of discipline or motivation (n = 3) [32, 34, 36]. Two studies cited a limited exercise history or a sedentary lifestyle [32, 50], whereas one cited exercise as boring and unenjoyable [50]. Significant life stressors (e.g., funerals) [45] and unemployment [45] also reduce motivation and lead individuals to deprioritize exercise.

### Facilitators

Ten facilitators were identified. Having a history of exercise engagement (n = 4) [40, 45, 46, 50] or previously experiencing positive benefits (n = 4) [40, 49, 50, 54], such as regaining strength or improved emotional well-being, were the most prominent facilitators. Similarly, perceiving exercise as beneficial (n = 3), regardless of prior experience, also promoted engagement [46, 47, 49]. In contrast, the shock of diagnosis provided a teachable moment in three studies to promote exercise [32, 36, 47]. High internal drive (n = 2) [35, 49] and perceptions of exercise as enjoyable (n = 3) [36, 46, 50], achievable (n = 2) [46, 49], and safe (n = 1) [49] also facilitated engagement. Some participants who experienced physical deterioration adopted exercise to counter decline (n = 1) [45] and maintain daily activities (n = 1) [50], particularly when living alone.

### Theme 3: Program design and delivery determinants of engagement

This theme relates to elements of exercise program design and delivery, such as exercise setting, resource availability, exercise scheduling and the mode of facilitation, i.e., one-to-one or group-based exercise.

### Barriers

Eleven barriers were identified, with travel or distance to attend programs (n = 5) [40, 41, 43, 46, 50] most frequently cited, as these barriers were considered time-consuming and contributed to fatigue. Other barriers included adverse weather, particularly if participants were required to travel to an exercise location, or if they were outdoors, and home-based exercises, such as walking (n = 3), were included as part of the program [45, 46, 50]. A lack of resources or facilities (n = 3) [47, 51, 54], particularly when associated with financial costs (n = 1) [50], also limits exercise engagement. The exercise setting also posed challenges. For example, hospital-based programs [35] are difficult when daily sessions are prescribed because of added financial costs, time demands, and fatigue. Conversely, home-based exercise [32], especially unsupervised [37, 43] or performed alone without a partner or group [32], was linked to low motivation, lack of accountability, and no real-time feedback. Two studies (n = 2) reported technology use (e.g., computers or smartphones) as a barrier to exercise delivery due to personal disliking and technical difficulties such as poor internet connectivity [41, 50]. Prescribing fixed exercise intensities and limited variety (n = 1) [53] were also cited as barriers.

### Facilitators

Twenty facilitators were identified, with personalized programs (n = 13) being the most prominent. Personalization involves strategies such as individualized goal setting and tailoring to participant preferences [32, 33, 35–37, 44, 49–55]. Adaptable programs (n = 4) [37, 51, 53, 54] and those incorporating exercise monitoring and progression (n = 6) [35, 37, 40, 49, 50, 52] have also supported engagement. Home- or community-based programs were also cited (n = 7), offering convenient and accessible exercise locations [33–35, 37, 39, 47]. Two studies highlighted convenience as key [46, 47]. In addition, hospital-based exercise (n = 2) was viewed as safe and supportive [47, 50], which is consistent with one study emphasizing addressing safety concerns as a facilitator [51]. Flexible scheduling of exercise sessions was also prominent in enhancing engagement (n = 6) [35, 36, 39, 46, 49, 50].

Six studies identified group-based exercise as a facilitator [32, 36, 40, 50, 51, 54], whereas five cited supervised exercise delivered in person or online [36, 37, 39, 41, 43], underscoring the value of social interaction and professional oversight. Two studies specifically highlighted virtual exercise as a facilitator, indicating that remote formats support engagement [41, 43].

Additional facilitators included light-intensity exercise [47, 53], the absence of specialist equipment [35, 46], and exercise variety [45]. The optimized exercise duration was cited [36, 49], although the optimal length varied between studies, for example, 12–14 weeks [36] vs. six weeks [49]. Regardless, programs perceived as neither too short nor too long were preferred. One study [45] reported that activities emphasizing strategy or technique aided adherence by providing a distraction from the physical demands of exercise. Treatment-specific programs [48, 51] where exercise modality and setting were adapted to treatment regimens (e.g., home-based for weekly chemotherapy, hospital-based for daily radiotherapy) were also cited. The incorporation of exercise into multimodal programs that address broader holistic needs (e.g., nutrition) [37, 51] and provide clear, practical, and affirming educational materials [49, 51, 54] also supported exercise engagement.

### Theme 4: Practical, logistic and interpersonal factors

This theme captures the practical considerations experienced by individuals with LC when engaging in exercise during treatment.

### Barriers

Four barriers were identified, with treatment scheduling being the most prominent (n = 6) [32, 34, 37, 41, 43, 48], as frequent or prolonged appointments reduced time and energy for exercise. A lack of time from nontreatment commitments was cited in two studies [41, 50], with one specifically noting employment-related fatigue [50]. Three studies [36, 45, 50] also reported a lack of peer support, where family or friends discouraged exercising due to perceived harm and instead promoted rest.

### Facilitators

Eight facilitators were identified, with peer support (n = 10) [35–37, 45–47, 49, 51, 52, 54] and professional support (n = 9) [33, 35–37, 45, 49, 50, 52, 54] being the most prominent. Peer support encouraged and motivated participants, whereas professional support offered reassurance. Carer support was cited as a facilitator [51], alongside trust in professionals (n = 2) [32, 54] and professional advocacy (n = 1) [54]. These findings highlight the importance of affirming messaging from healthcare providers and diverse support systems to promote engagement. Aligning exercise with treatment appointments was also cited, as it provided practical convenience and reduced hospital visits (n = 3) [41, 42, 49]. Adopting accountability measures, such as sending regular reminders via text messages or phone calls, also supported adherence (n = 4) [35, 43, 50, 54]. Finally, one cohort study [48] reported cultural preferences for active transport, such as walking or cycling, as facilitators of exercise engagement.

### Theme 5: Professional and clinical considerations

This theme captures factors related to the clinical management of LC, as well as specific influencing factors expressed by HCPs.

### Barriers

Five barriers emerged, with cancer treatment most frequently cited as a limiting factor (n = 8) [40, 41, 43, 44, 51, 52, 54, 55]. While the reasons are not always explicit, some studies reported side effect exacerbation at specific points in treatment cycles or as treatment progressed, discouraging exercise [51]. Comorbidities were also barriers in six studies [45, 46, 50, 51, 53, 55], with one citing medication side effects [52]. Two qualitative studies involving HCPs identified limited referral pathways [51, 54] and high clinical workloads [51], highlighting organizational constraints and limited access to exercise services, reducing the capacity of HCPs to promote or facilitate exercise during treatment.

### Facilitators

No facilitators were identified within this theme.

### Exercise-related adverse events

Among the thirteen studies incorporating an exercise program, eight (n = 8) included data on exercise-related adverse events [31, 33–37, 39, 43], and five (n = 5) did not [32, 38, 40–42]. Two studies reported fatigue and muscle soreness as minor adverse events (n = 2) [31, 43]. No major exercise-related adverse events were reported [31, 33–37, 39, 43].

## Discussion

This ScR mapped 31 barriers and 42 facilitators, organized into five themes. Exercise remains a critical component of cancer care, as evidence continues to emerge demonstrating its benefits, including improved survival in colorectal cancer survivors [57]. In LC, recent reviews [17, 21] acknowledge the potential of exercise as an adjuvant therapy, underscoring the need for clinical trials to confirm its effects. However, research on tailoring LC-specific exercise programs remains limited [58]. Therefore, addressing this gap is essential for improving LC survivor engagement in exercise research during cancer treatment to enable robust efficacy trials [17, 21]. This ScR contributes to this gap by mapping the barriers and facilitators that people with LC experience when exercising throughout cancer treatment.

### Barriers and facilitators

LC and its treatment are associated with symptoms and side effects that impair physical functioning [21, 55]. This review identified several treatment-related symptoms that act as barriers to exercise engagement, most commonly fatigue, pain or discomfort, dyspnea, and increased infection risk. Evidence from this ScR also suggests that side effects often intensify at specific points within treatment cycles or as treatment progresses, further limiting exercise engagement. This is a well-documented phenomenon, particularly during RT, as symptoms typically peak in the weeks following treatment completion [59].

Treatment scheduling was also identified as a barrier to exercise. For example, carboplatin and paclitaxel with concurrent radiotherapy, a common LC treatment, requires weekly chemotherapy and daily RT for four to seven weeks [60], necessitating frequent hospital visits. The evidence from this ScR suggests that these scheduling demands impose substantial time and energy burdens on patients, underscoring the need for treatment-specific exercise programs with ongoing professional support to manage symptoms and side effects. Practical, logistical, interpersonal, and intrapersonal factors must also be considered to ensure that exercise programs during LC treatment remain adaptable to treatment schedules and individuals’ circumstances.

In addition, personalization was frequently cited as a facilitator. To achieve personalization, recent research protocols highlight the utility of codesign and patient and public involvement in research (PPIR) as effective strategies [61]. For example, Wade-McBane [61] implemented codesign methods by collaborating with LC survivors and HCPs to identify priorities for an LC prehabilitation program. Similarly, Toohey [21] recommended multidisciplinary team integration to ensure that exercise interventions align with treatment goals and individual holistic needs. The incorporation of behavior change techniques, such as personalized goal setting and providing education, has also been used as a strategy to achieve exercise personalization [21, 61]. The facilitators identified within the ScR, such as the provision of clear and practical educational materials in addition to facilitating physical activity counseling, further reinforce the value of these approaches.

However, some barriers and facilitators identified in this review are contradictory. For example, hospital-based exercise and home-based exercise were each reported as barriers to and facilitators of exercise, reflecting divergent personal preferences. Similarly, while exercise variety was identified as a facilitator, limited resources were cited as a barrier, indicating that the capacity to personalize exercise programs may be limited by resource availability.

Finally, with the exception of two minor adverse events, no serious exercise-related adverse events were reported, aligning with ASCO’s position [8] that exercise during cancer treatment carries a low risk when appropriate medical screening and tailored prescriptions are implemented.

### Study limitations

The inclusion of studies from multiple countries introduces heterogeneity, as healthcare systems vary between countries, limiting the universal applicability of some barriers and facilitators. Participant characteristics also vary widely, from early- to advanced-stage LC, with minimal representation of SCLC, making stage- or subtype-specific insights unclear. Furthermore, only two studies [39, 42] were adequately powered; therefore, there is limited availability of sufficiently powered data to support recommendations.

### Recommendations

On the basis of the ScR results, the following recommendations may assist researchers and HCPs in creating exercise programs for people with LC during treatment:

1. A multidisciplinary approach is adopted for the design and delivery of exercise programs during treatment to address the holistic needs of individuals, including nutrition and mental health. This approach also facilitates timely management of symptoms and side effects, thereby supporting exercise adherence.
2. Collaborate with HCPs and individuals affected by LCs, including friends and family members, to codesign exercise programs that are tailored to treatment protocols and personal circumstances, ensuring that sessions are scheduled to accommodate treatment cycles and hospital appointments.
3. Baseline assessments of fitness, psychological status, and personal circumstances should be conducted to guide goal setting and personalized program design. Where appropriate, family or friends should be involved in initial sessions to reduce anxiety and strengthen support.
4. Provide clear, practical education on the benefits of exercise during treatment and incorporate ongoing physical activity counseling throughout the program.
5. Flexible exercise programs that allow adjustments to intensity, duration, and modality on the basis of performance and preferences should be designed, and tracking methods should be used to monitor progress and guide modifications.
6. Provide regular professional supervision alongside or independent of unsupervised exercise to ensure safety, foster accountability, and build trust while offering participants and family members support and reassurance.
7. Facilitate peer support, such as occasional group-based sessions or encouraging family involvement in exercise activities, to increase motivation and reduce intrapersonal barriers.

## Conclusion

Despite its potential benefits, engaging people with LC in exercise programs during cancer treatment poses significant challenges, limiting research progress and service delivery [18, 22]. However, leveraging the facilitators and recommendations identified in this ScR, such as stakeholder collaboration through codesign, can inform the development of patient-centered, high-quality exercise research and services that encourage the engagement of LC survivors.

## Supplementary Information

Below is the link to the electronic supplementary material.

Supplementary file1 (DOCX 26 KB)

Supplementary file2 (DOCX 19 KB)

Supplementary file3 (XLSX 2561 KB)

## Data Availability

All data supporting the findings of this study are available within the paper and its supplementary information, referenced according to the journal as ‘online resources’. The PRISMA-SCR checklist is provided in online resource 1. The search strategy is provided in online resource 2. The data extraction sheet, which includes the initially extracted codes, justification for their refinement, and grouping within themes, is provided as online resource 3. Table 1 provides the details of all the papers included for data extraction. Table 2 summarizes the research methodologies of the included studies. Table 3 summarizes the intervention characteristics of the included studies that involved an exercise program. Finally, Fig. 1 provides the PRISMA flow diagram, tracking the search process and providing reasons for exclusion. The provision of this information ensures the complete transparency of research.
