# Supplementary material for: Barriers and facilitators to exercise engagement during lung cancer treatment: a scoping review
Source: Cancer Causes Control. 2026 May 26;37(6):95. doi: 10.1007/s10552-026-02177-6 (PMC13212711; doi:10.1007/s10552-026-02177-6)
Supplement: Supplementary file 2 — Supplementary file2 (DOCX 19 KB) [file 10552_2026_2177_MOESM2_ESM.docx]

Online resource 2

**Article title:** Barriers and Facilitators to Exercise Engagement During Lung Cancer Treatment: A Scoping Review

**Journal name:** Supportive Care in Cancer

**Author names:** Matthew Beggs, Gillian Prue, Joanne Reid

**Affiliation:** Queens University Belfast

**E-mail address of corresponding author:** mbeggs06@qub.ac.uk

Search Strategy

**Concept 1:**  Physical Activity

Physical activity *‘*OR’ Physical exercise ‘OR’ Movement ‘OR’ Exercise* ‘OR’ aerobic ‘OR’ cycling ‘OR’ cycle ‘OR’ walk* *‘*OR’ resistance training *‘*OR’ pre-habilitation *‘*OR’ prehabilitation *‘*OR’ prehab *‘*OR’ pre-hab *‘*OR’ aerobic capacity *‘*OR’ exercise tolerance *‘*OR’ High-Intensity Interval Training *‘*OR’ HIIT *‘*OR’ Re-HIIT *‘*OR’ Low-intensity *‘*OR’ moderate intensity *‘*OR’ biking *‘*OR’ lifestyle intervention *‘*OR’ low-impact *‘*OR’ high impact *‘*OR’ exercise therapy *‘*OR’ run *‘*OR’ running *‘*OR’ pulmonary rehab* *‘*OR’ strength *‘*OR’ exercise training *‘*OR’ vigorous *‘*OR’ activities of daily living *‘*OR’ activity *‘*OR’ exercise capacity *‘*OR’ pre-operative *‘*OR’ perioperative *‘*OR’ physical therapy *‘*OR’ ADL

**Concept 2:**  Lung Cancer

Lung cancer *‘*OR’ lung neoplasm *‘*OR’ lung tumo* *‘*OR’ lung adenocarcinoma *‘*OR’ non-small cell lung carcinoma *‘*OR’ NSCLC *‘*OR’ SCLC *‘*OR’ small-cell lung cancer *‘*OR’ thoracic neoplasm *‘*OR’ thoracic cancer *‘*OR’ thoracic carcinoma *‘*OR’ lung nodules *‘*OR’ mesothelioma *‘*OR’ large cell carcinoma *‘*OR’ squamous cell carcinoma *‘*OR’ small-cell lung carcinoma

**Concept 3:**  Barriers and Facilitators

Barrier* *‘*OR’ facilitate* *‘*OR’ adherence *‘*OR’ recruit* *‘*OR’ difficult* *‘*OR’ challenge* *‘*OR’ implement* *‘*OR’ motivate* *‘*OR’ enable *‘*OR’ encourage *‘*OR’ discourage *‘*OR’ obstacle *‘*OR’ impact *‘*OR’ compliance

**Search between concepts**

Concept 1 ‘AND’ Concept 2 ‘AND’ Concept 3

**Additional limits**

Humans

Full-text

Adults (aged 18+)

English language

* = Truncated word

**Additional Searches**

- Hand-picked research articles which were not identified within the initial searches, including grey literature, for example: conference papers from relevant organisations such as Multinational Association of Supportive Care in Cancer (MASCC) or the British Thoracic Oncology Group (BTOG); and Screening of Queens University Belfast online theses library.
- Hand-picked articles identified through ‘Snowball’ searching of reference lists

**EBSCO Host**

S1 - AB (lung cancer or lung neoplasm* or lung tumor or lung adenocarcinoma OR lung tumour)

S2 - AB (nsclc or non small cell lung cancer OR small cell lung carcinoma)

S3 - AB (sclc OR small cell lung cancer OR small cell lung carcinoma)

S4 - AB (thoracic neoplasm OR thoracic cancer OR thoracic carcinoma)

S5 – S1 OR S2 OR S3 OR S4

S6 - AB pulmonary disease

S7 - (MH "Pulmonary Disease, Chronic Obstructive+")

S8 - AB (chronic obstructive pulmonary disease* OR TI chronic obstructive pulmonary disease*)

S9 - AB (chronic obstructive lung disease* OR TI chronic obstructive lung disease*)

S10 - AB (chronic obstructive airway disease* OR TI chronic obstructive airway disease*)

S11 - AB (chronic airflow obstruction OR TI chronic airflow obstruction)

S12 - AB (chronic bronchitis OR TI chronic bronchitis)

S13 - AB (emphysema OR TI emphysema)

S14 - AB (COPD OR TI COPD)

S15 - AB (interstitial lung disease or pulmonary fibrosis)

S16 - S6 OR S7 OR S8 OR S9 OR S10 OR S11 OR S12 OR S13 OR S14 OR S15

S17 - AB ((MH "Exercise+") OR "exercise" OR (MH "Exercise Tolerance+"))

S18 - AB ((MH "Aerobic Exercises") OR (MH "Aerobic Capacity") OR "aerobic exercise" OR (MH "Anaerobic Exercises") OR (MH "Exercise Tolerance") OR (MH "Resistance Training") OR (MH "Prehabilitation"))

S19 - S17 OR S18

S20 - TX (barrier* or obstacle* or difficult* or challenge* or issue* or problem*)

S21 - TX implementation

S22 - TX (strategy or strategies or methods or techniques)

S23 - TX (facilitate OR facilitators or motivator* or enabler*)

S24 - S20 OR S21 OR S22 OR S23

S25 - S5 OR S16

S26 - S19 AND S24 AND S25

**Pubmed**

**#1** - "lung cancer"[Title/Abstract] OR "lung neoplasm*"[Title/Abstract] OR "lung tumor"[Title/Abstract] OR "lung adenocarcinoma"[Title/Abstract] OR "lung tumour"[Title/Abstract]

**#2** - "nsclc"[Title/Abstract] OR "non small cell lung cancer"[Title/Abstract] OR "small cell lung carcinoma"[Title/Abstract]

**#3** - "sclc"[Title/Abstract] OR "small cell lung cancer"[Title/Abstract] OR "small cell lung carcinoma"[Title/Abstract]

**#4** - "thoracic neoplasm"[Title/Abstract] OR "thoracic cancer"[Title/Abstract] OR "thoracic carcinoma"[Title/Abstract]

**#5** - "lung cancer"[Title/Abstract] OR "lung neoplasm*"[Title/Abstract] OR "lung tumor"[Title/Abstract] OR "lung adenocarcinoma"[Title/Abstract] OR "lung tumour"[Title/Abstract] OR "nsclc"[Title/Abstract] OR "non small cell lung cancer"[Title/Abstract] OR "small cell lung carcinoma"[Title/Abstract] OR "sclc"[Title/Abstract] OR "small cell lung cancer"[Title/Abstract] OR "small cell lung carcinoma"[Title/Abstract] OR "thoracic neoplasm"[Title/Abstract] OR "thoracic cancer"[Title/Abstract] OR "thoracic carcinoma"[Title/Abstract]

**#6** - "lung diseases"[MeSH Terms] OR "pulmonary disease"[Title/Abstract] OR "chronic obstructive pulmonary disease*"[Title/Abstract] OR "chronic obstructive pulmonary disease*"[Title/Abstract] OR "chronic obstructive lung disease*"[Title/Abstract] OR "chronic obstructive lung disease*"[Title/Abstract] OR "chronic obstructive airway disease*"[Title/Abstract] OR "chronic obstructive airway disease*"[Title/Abstract] OR "chronic airflow obstruction"[Title/Abstract] OR "chronic airflow obstruction"[Title/Abstract] OR "chronic bronchitis"[Title/Abstract] OR "chronic bronchitis"[Title/Abstract] OR "emphysema"[Title/Abstract] OR "emphysema"[Title/Abstract] OR "COPD"[Title/Abstract] OR "COPD"[Title/Abstract] OR "interstitial lung disease"[Title/Abstract] OR "pulmonary fibrosis"[Title/Abstract]

**#7** - "exercise"[Title/Abstract] OR "aerobic"[Title/Abstract] OR "aerobic exercise"[Title/Abstract] OR (("anaerob"[All Fields] OR "anaerobic"[All Fields] OR "anaerobically"[All Fields] OR "anaerobics"[All Fields] OR "anaerobs"[All Fields] OR "bacteria, anaerobic"[MeSH Terms] OR ("bacteria"[All Fields] AND "anaerobic"[All Fields]) OR "anaerobic bacteria"[All Fields] OR "anaerobe"[All Fields] OR "anaerobes"[All Fields]) AND ("exercise"[MeSH Terms] OR "exercise"[All Fields] OR "exercises"[All Fields] OR "exercise therapy"[MeSH Terms] OR ("exercise"[All Fields] AND "therapy"[All Fields]) OR "exercise therapy"[All Fields] OR "exercising"[All Fields] OR "exercise s"[All Fields] OR "exercised"[All Fields] OR "exerciser"[All Fields] OR "exercisers"[All Fields])) OR (("aerobic"[All Fields] OR "aerobically"[All Fields] OR "bacteria, aerobic"[MeSH Terms] OR ("bacteria"[All Fields] AND "aerobic"[All Fields]) OR "aerobic bacteria"[All Fields] OR "aerobe"[All Fields] OR "aerobes"[All Fields] OR "exercise"[MeSH Terms] OR "exercise"[All Fields] OR "aerobics"[All Fields]) AND ("capacities"[All Fields] OR "capacity"[All Fields])) OR ("exercise tolerance"[MeSH Terms] OR ("exercise"[All Fields] AND "tolerance"[All Fields]) OR "exercise tolerance"[All Fields]) OR ("resistance training"[MeSH Terms] OR ("resistance"[All Fields] AND "training"[All Fields]) OR "resistance training"[All Fields]) OR ("prehabilitative"[All Fields] OR "preoperative exercise"[MeSH Terms] OR ("preoperative"[All Fields] AND "exercise"[All Fields]) OR "preoperative exercise"[All Fields] OR "prehabilitation"[All Fields] OR "pre-hab"[All Fields] OR "prehab"[All Fields] OR "pre-habilitation"[All Fields])

**#8** - "barrier*"[All Fields] OR "obstacle*"[All Fields] OR "difficult*"[All Fields] OR "challenge*"[All Fields] OR "issue*"[All Fields] OR "problem*"[All Fields] OR "implementability"[All Fields] OR "implementable"[All Fields] OR "implementation"[All Fields] OR "implementation s"[All Fields] OR "implementational"[All Fields] OR "implementations"[All Fields] OR "implementer"[All Fields] OR "implementers"[All Fields] OR "implemention"[All Fields] OR "strategie"[All Fields] OR "strategies"[All Fields] OR "strategy"[All Fields] OR "strategy s"[All Fields] OR "strategie"[All Fields] OR "strategies"[All Fields] OR "strategy"[All Fields] OR "strategy s"[All Fields] OR "method s"[All Fields] OR "methods"[MeSH Terms] OR "methods"[All Fields] OR "method"[All Fields] OR "methods"[MeSH Subheading] OR "methods"[MeSH Terms] OR "methods"[All Fields] OR "technique"[All Fields] OR "methods"[MeSH Subheading] OR "techniques"[All Fields] OR "technique s"[All Fields] OR "facilitate"[All Fields] OR "facilitated"[All Fields] OR "facilitates"[All Fields] OR "facilitating"[All Fields] OR "facilitation"[All Fields] OR "facilitations"[All Fields] OR "facilitative"[All Fields] OR "facilitator"[All Fields] OR "facilitator s"[All Fields] OR "facilitators"[All Fields] OR "facilitate"[All Fields] OR "facilitated"[All Fields] OR "facilitates"[All Fields] OR "facilitating"[All Fields] OR "facilitation"[All Fields] OR "facilitations"[All Fields] OR "facilitative"[All Fields] OR "facilitator"[All Fields] OR "facilitator s"[All Fields] OR "facilitators"[All Fields] OR "motivate"[All Fields] OR "motivated"[All Fields] OR "motivates"[All Fields] OR "motivating"[All Fields] OR "motivation"[MeSH Terms] OR "motivation"[All Fields] OR "motivations"[All Fields] OR "motive"[All Fields] OR "motivational"[All Fields] OR "motivator"[All Fields] OR "motivators"[All Fields] OR "motives"[All Fields] OR "motivate"[All Fields] OR "motivated"[All Fields] OR "motivates"[All Fields] OR "motivating"[All Fields] OR "motivation"[MeSH Terms] OR "motivation"[All Fields] OR "motivations"[All Fields] OR "motive"[All Fields] OR "motivational"[All Fields] OR "motivator"[All Fields] OR "motivators"[All Fields] OR "motives"[All Fields] OR "enable*"[All Fields]

**Ovid**

**1** - (lung* and (cancer* or neoplasm* or carcinoma*)).ti,ab.

**2** - (small cell lung* and (cancer* or carcinoma*)).ti,ab.

**3** - (thoracic* and (neoplasm* or cancer* or carcinoma*)).ti,ab.

**4** - pulmonary disease.ti,ab.

**5** - (Chronic obstructive* and (pulmonary disease* or airway disease*)).ti,ab.

**6** - chronic bronchitis.ti,ab.

**7** - emphysema.ti,ab.

**8** - COPD.ti,ab.

**9** - interstitial lung disease.ti,ab.

**10** - pulmonary fibrosis.ti,ab.

**11** - 1 or 2 or 3

**12** - 4 or 5 or 6 or 7 or 8 or 9 or 10

**13** - 11 or 12

**14** - exercise.ti,ab.

**15** - exercise tolerance.ti,ab.

**16** - aerobic exercise.ti,ab.

**17** – aerobic capacity.ti,ab.

**18** – aerobic exercise.ti,ab.

**19** – anaerobic exercise.ti,ab.

**20** - exercise tolerance.ti,ab.

**21** - resistance training.ti,ab.

**22** - prehabilitation.ti,ab.

**23 -** prehab.ti,ab.

**24** - prehab.ti,ab.

**25 -** pre-habilitation.ti,ab.

**26** - 14 or 15 or 16 or 17 or 18 or 19 or 20 or 21 or 22 or 23 or 24 or 25

**27** - (barrier$ or obstacle$ or difficult$ or challenge$ or issue$ or problem$ or implementation or strateg$ or methods or techniques or facilitat$ or motivator$ or enable$), ab, tx, ct, sh, hw, tn, ot, dm, mf, dv, kf, fx, dq, rw, nm, bt, ox, px, rx, an, ui, sy, ux, mx, id, tc, tm, pt]

**28** - 13 and 26 and 27
